# The Odontographic Society of Chicago

**Published:** 1898-03

**Authors:** 


### EDITORIAL.

### The Odontographic Society of Chicago.

The annual meeting of this body was held in Chicago February 21st and 22d. The meeting was largely attended and was quite interesting. It consisted largely of clinics and demonstrations, in which many new, interesting and valuable methods, appliances and preparations were presented. The clinic was a very large one and would require pages to give a full description of it.

There were present, including members and visitors, between four and five hundred persons, and the time was very fully occupied, and a large number of persons took part in the work of the occasion. Many things out of the ordinary course were presented, and fully illustrated and demonstrated.

The sessions were held the first day in the North-western University Dental School, on the second day in the Chicago College of Dental Surgery, each of which afforded ample facilities for all the work to be done.

Papers were read and discussed on the first evening by Drs. Black, Harlan and Taft. On the second evening a banquet was given by the society at the Palmer House, at which there was a splendid social time, highly enjoyed and appreciated by every member present.

This society has had a phenomenal growth. Organized about seven years ago, with, perhaps, half a dozen members, it has grown in the meantime to a membership of over two hundred and fifty. At this meeting one hundred and fifty new members were brought in, making the present membership over four hundred. It is the largest association in the country, except, perhaps, the *National*. This growth is characteristic of Chicago.

This organization is an illustration of what may be done in any town, city or district having a considerable number of dentists, in the way of organization. A change from the ordinary method from that of chiefly scientific work to that of a more practical nature, seemed to be highly appreciated by the larger proportion of those present. This line of work has undoubtedly, in the past, been too much overlooked and neglected, but it is to be hoped that a permanent change is taking place in this respect, which change may be very profitably adopted by nearly all dental societies.

There is, perhaps, less room and opportunity for such work in our State and National Societies, but all others may adopt this practical line with very great benefit.

The concensus of expression of all present was, that this meeting of the Odontographic Society of Chicago was an eminent success.

Doubtless this body is to have a career of great usefulness. Long may it live.

